# A case of primary gastric undifferentiated high-grade pleomorphic sarcoma diagnosed with chief complaint of fever: a case report and literature review

**DOI:** 10.1186/s40792-017-0317-z

**Published:** 2017-03-08

**Authors:** Akira Kabashima, Koichi Kimura, Kensaku Sanefuji, Satoru Masunari, Seiji Haraoka, Soichiro Maekawa

**Affiliations:** 1 Department of Surgery, Munakata Medical Association Hospital, 5-3-3 Taguma, Munakata, Fukuoka 811-3431 Japan; 2 Department of Radiology, Munakata Medical Association Hospital, 5-3-3 Taguma, Munakata, Fukuoka 811-3431 Japan; 3 Department of Pathology, Fukuoka University Chikushi Hospital, Chikushino, Fukuoka 818-0067 Japan

**Keywords:** Undifferentiated high-grade pleomorphic sarcoma, Stomach, Fever, Granulocyte colony-stimulating factor (G-CSF)

## Abstract

**Background:**

Undifferentiated high-grade pleomorphic sarcoma in gastrointestinal tract is extremely rare, and its prognosis is poor.

**Case presentation:**

An 82-year-old man visited a previous hospital complaining of fever, general fatigue, and shaking chill, for which he received antibiotics therapy. As the fever continued, he was referred to our hospital, where computed tomography and upper gastrointestinal endoscopy showed a 6-cm gastric tumor. A preoperative biopsy was consistent with a malignant mesenchymal tumor, but could not provide a definitive pathological diagnosis nor prove a cause-and-effect relationship between the chief complaint and the gastric tumor. The gastric tumor had grown to 8 cm in diameter within a month so we performed a partial gastrectomy. The pathological postoperative diagnosis was undifferentiated high-grade pleomorphic sarcoma that produced granulocyte colony-stimulating factor. The patient’s fever quickly improved, and he showed a good postoperative course.

**Conclusions:**

We herein report a case of rapidly growing, undifferentiated, high-grade pleomorphic gastric sarcoma, which presented as a chief complaint of fever.

## Background

Undifferentiated high-grade pleomorphic sarcoma (UPS) is a soft-tissue tumor usually found in the limbs and in the retroperitoneum. Its prognosis is poor. Occurrence in gastrointestinal tract is extremely rare. Here, we report a rare case of rapidly growing gastric UPS associated with a persistent fever and discuss the relevant literature.

## Case presentation

**Fig. 1:**
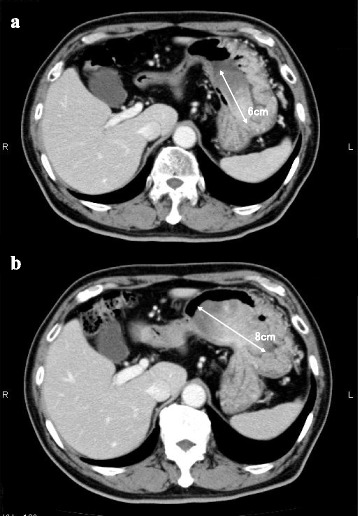
Abdominal computed tomography (CT) showed a 6-cm gastric tumor in early August (**a**) that had enlarged to 8 cm in diameter by early September (**b**)

**Fig. 2:**
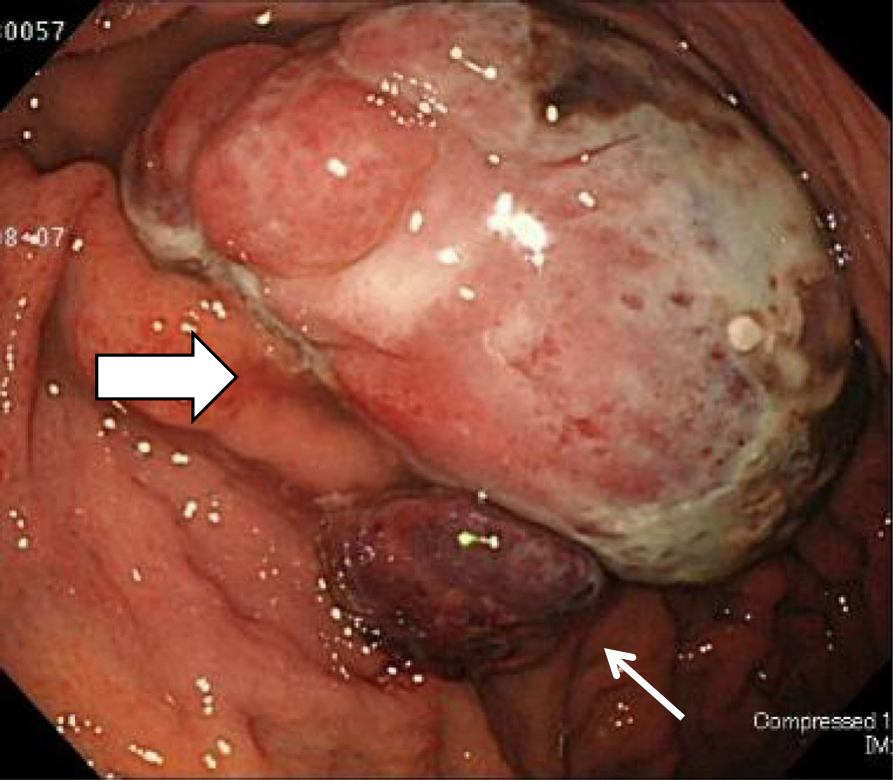
Gastrointestinal endoscopy in early August showed a 6-cm pedunculated tumor on the greater curvature in the gastric body (*thick arrow*); nearby, a 2-cm sub-pedunculated tumor was also observed (*thin arrow*)

An 82-year-old man presented at a nearby hospital with fever, general fatigue, and shaking chill in July 2015. Although he was treated with antibiotics for his high level of c-reactive protein (CRP), his 38–39 °C fever remained. He was therefore referred to our hospital. When first examined, his body temperature was 38.4 °C but had normal blood pressure and pulse rate. No abnormal palpable mass was reported. His blood examination findings showed high inflammatory reaction, slightly low coagulability, and mildly activated fibrinolysis. However, we observed no immunoglobulin or specific antibody that could be considered the cause of his fever (Table 1). Abdominal computed tomography (CT) in early August showed a 6-cm tumor in the stomach (Fig. 1a) with an estimated volume of 18.07 ml. Neither other tumor nor abscess was observed. Gastrointestinal endoscopy (GIE) in early August showed a 6-cm pedunculated oval tumor on the upper part of the greater gastric curvature (Fig. 2). We also found a nearby 2-cm sub-pedunculated oval tumor. They were elastic-hard, with a smooth surface and a sparse ductal structure.

**Table 1:** Patient’s clinical data

Blood count		Blood chemistry		Immune-related tests	
WBC	8300/μl	TP	6.9 g/dl	IgG	1683 mg/dl
RBC	384/μl	Alb	3.0 g/dl	IgA	243 mg/dl
Hb	10.8 g/dl	GOT	18 U/l	IgM	132 mg/dl
Ht	35.7%	GPT	15 U/l	Anti SS-A/Ro	<7.0 U/ml
Plt	22.5 × 10^4^/μl	LDH	139 U/l	Anti SS-B/La	<7.0 U/ml
Clotting factors		ALP	279 U/l	β-D-glucan	7.5 pg/ml
PT	13.1 s	γ-GTP	52 U/l	MMP-3	84.3 ng/ml
PT-INR	1.23	BUN	15.2 mg/dl	PR3-ANCA	<1.0 U/ml
Fib	594.6 mg/dl	Cr	0.91 mg/dl	MPO-ANCA	<1.0 U/ml
FDP	6.6 μg/ml	AMY	60 U/l	sIL-2R	411 U/ml
D-dimer	1.2 μg/ml	Fe	44 mg/dl	ANA	<40 times
		CRP	11.20 mg/dl	Anti CCP	<0.6 U/ml
				Procalcitonin	0.07 ng/ml
				RF	<3 IU/ml

**Fig. 3:**
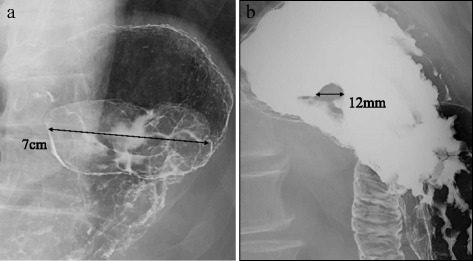
Upper gastrointestinal fluoroscopy in early August showed a 7-cm sub-pedunculated raised lesion (**a**) with a 12-mm attachment (**b**) on the greater curvature of the upper gastric body

Biopsy specimen showed the proliferation of atypical cells having pleomorphic or bizarre nuclei. Immunohistochemically, some atypical cells were positive for c-kit, alpha SMA, HHF-35, and calponin, but negative for EMA, cytokeratin (CK) CAM5.2, DOG-1, CD34, caldesmon, and desmin. Although these findings were consistent with malignant mesenchymal tumors, they did not confirm the pathological diagnosis. Upper gastrointestinal fluoroscopy in early August showed a 7-cm, multinodular sub-pedunculated raised lesions with a 12-mm attachment on the upper part of the greater gastric curvature (Fig. 3). The 2-cm tumor observed in the GIE was not visualized. In early September, a CT showed that the gastric tumor had expanded to 8 cm in diameter within the month, with the volume increased to 53.50 ml (Fig. 1b). The patient’s preoperative fever of 38 °C or higher, which was improved to 36 °C by taking acetaminophen, occurred once or twice every day. But, he had no symptoms such as abdominal pain and digestive tract obstruction.

We considered that surgery to patients with fever was dangerous, and we could not preoperatively confirm pathological diagnosis of the tumor or its cause-and-effect relationship to the main complaints. But, we finally chose to treat the tumor surgically because we found no lesion that could have caused the fever, and we were concerned that, if delayed, local resection of such a rapidly growing tumor would become impossible.

In mid-September, we performed a partial gastrectomy. We used laparoscopic assistance to disconnect the omentum from the cardia toward the gastric body to ensure mobility of the stomach. The upper stomach was then lifted out of the abdominal wall through a 6-cm incision in the left upper abdomen. A vertical incision was added from the cardia to the gastric body in the gastric anterior wall, through which both tumors were pulled outside, and their roots excised from stomach wall by automatic suturing devices (Endo-GIA with Tri-Stapler^,^ 30–2.5, Covidien), and then closed by Albert–Lembert sutures.

**Fig. 4:**
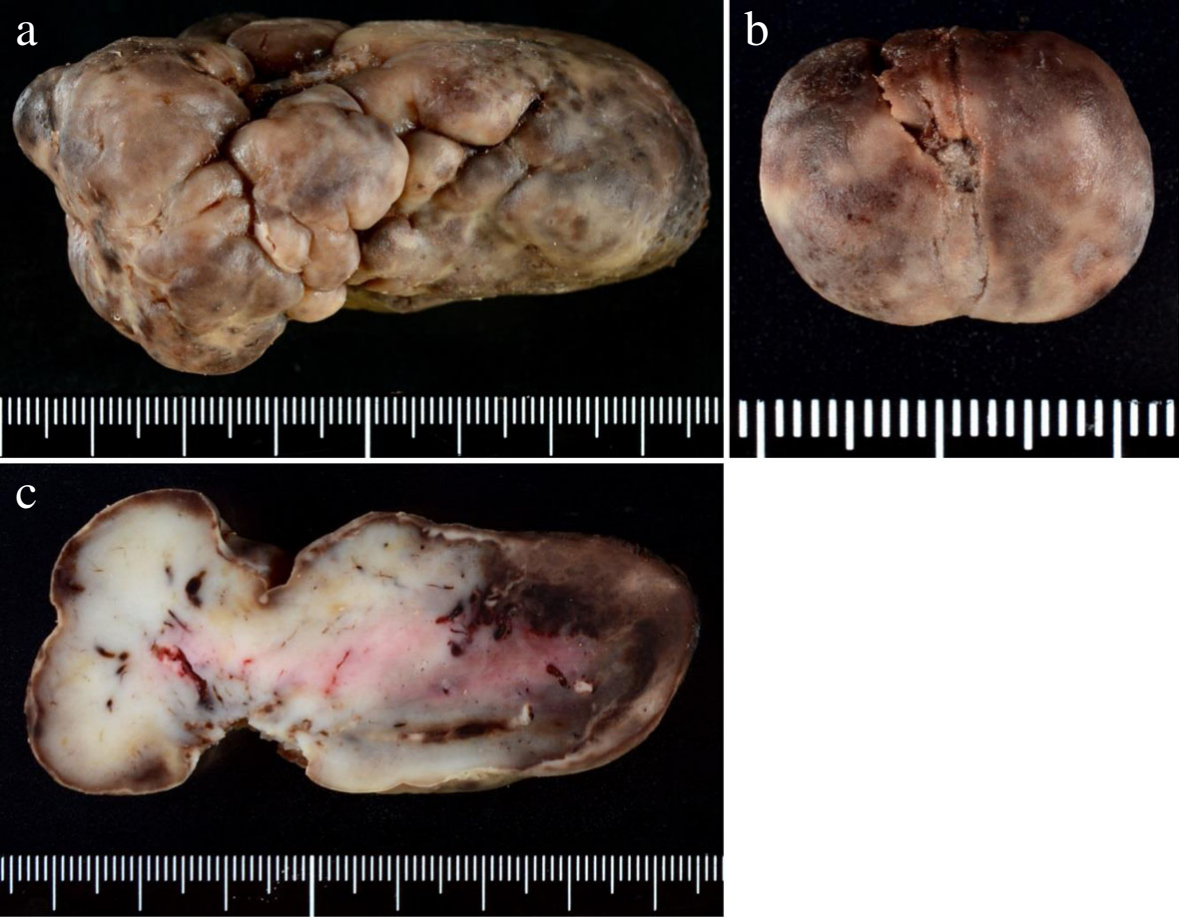
Gross findings of the tumors showed a pedunculated multinodular tumor (**a**) and a polypoid tumor (**b**). A *gray-white* tumor with focal hemorrhage was observed by cut surface of the large one (**c**). The tumor was observed very close to the surgical margin

**Fig. 5:**
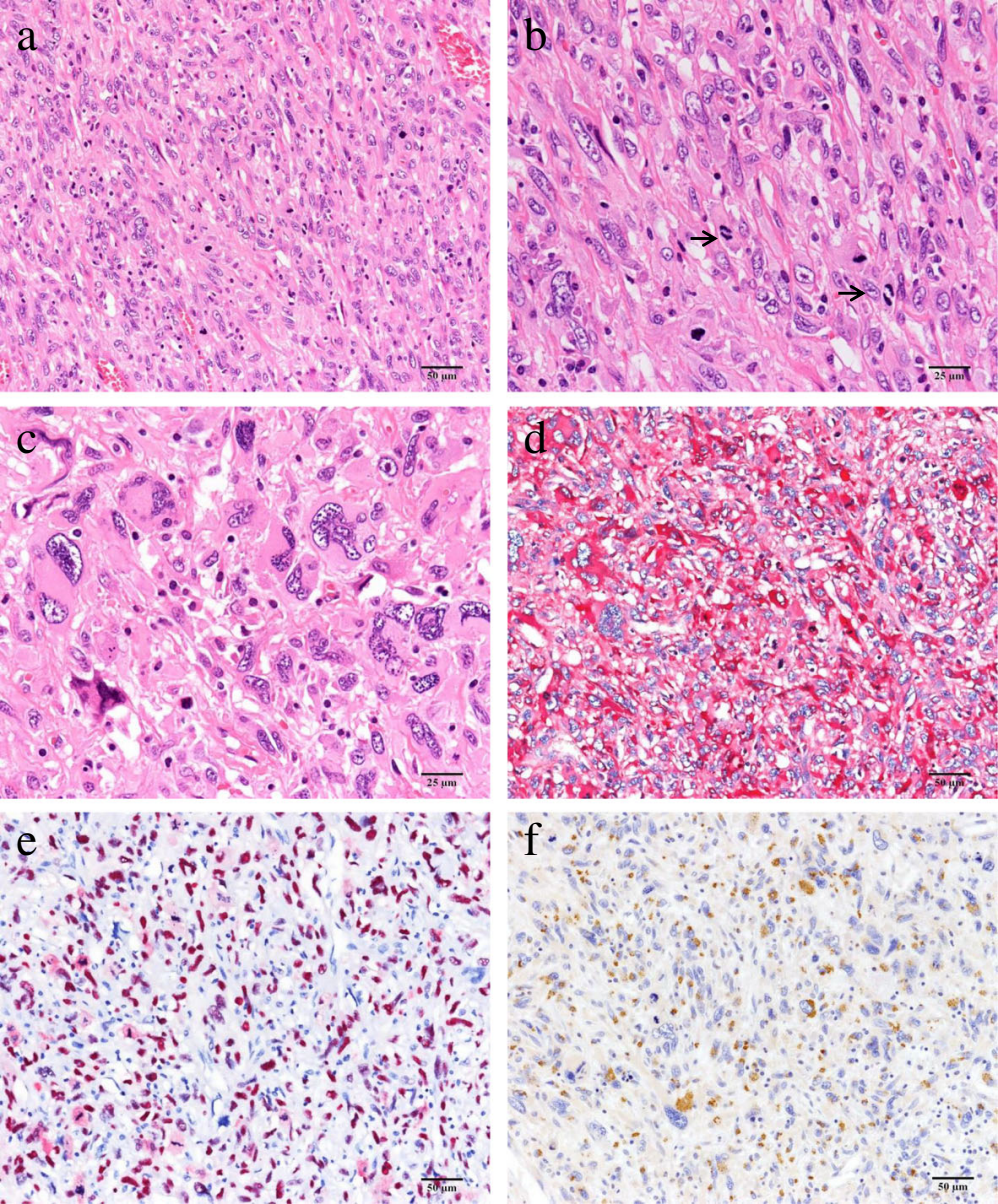
Microscopic findings showed proliferation of atypical spindle-shaped tumor cells arranged in sheets and irregular fascicles (**a**). Mitotic figures were frequently encountered (*arrows*, 20–25/10 HPF) and mild infiltration of lymphoplasmacytoid cells was noted (**b**). The large pleomorphic and bizarre tumor cells were also observed (**c**). Immunohistochemically, the tumor cells were positive for α1-antichymotrypsin (**d**), p-53 (**e**), and G-CSF (**f**)

Gross examination showed a pedunculated multinodular polypoid tumor (Fig. 4a) and a polypoid tumor (Fig. 4b), measured about 8 × 5 × 3.5 and 2.2 × 1.5 × 1 cm in size, respectively. Both tumors were solid-elastic consistency, smooth surface, and covered with ulcerated mucosa. The cut surface revealed the gray-white in color with foci of hemorrhage. Microscopically, the resected polypoid tumors were composed of atypical spindle cells and large pleomorphic or bizarre tumor cells arranged in sheets and irregular fascicles, associated with focal hemorrhage and surface ulceration. Mitotic figures (Fig. 5) including abnormal mitoses were frequently encountered (20–25/10 HPF). On immunohistochemical examination, tumor cells were positive for vimentin, α1-antichymotrypsin, CD68, CD163, G-CSF, p53, and Ki-67, some tumor cells were positive for alpha SMA, HHF35, and calponin, but tumor cells were negative for EMA, CK AE1/AE3, CK CAM5.2, c-kit, CD34, DOG1, desmin, caldesmon, S-100 protein, synaptophysin, hCG, HMB45, myoglobin, and CD30. The histologic and immunohistochemical features were consistent with undifferentiated high-grade pleomorphic sarcoma (pleomorphic malignant fibrous histiocytoma, UPS) producing granulocyte colony-stimulating factor (G-CSF).

Although the patient had a 38 °C fever twice on 1–2 postoperative days (POD), no fever or other complications were observed thereafter. He began taking meals on 5POD, and he was discharged with satisfactory progress on 12POD. Positron emission tomography (PET)/CT examination on 1 month after surgery showed accumulated fluoro-2-deoxy-D-glucose at the wound, but no other abnormal accumulation.

## Discussion

Malignant fibrous histiocytoma (MFH) has been classified into three subtypes: pleomorphic, inflammatory, and giant cell. By the World Health Organization (WHO) classification of bone and soft-tissue tumors of 2002, pleomorphic-type MFH was defined as a synonym for UPS [1]. Typically, UPS forms a large, rapidly growing solid mass and is histologically characterized by pleomorphic tumor cells in storiform patterns. UPS does not tend to show clear differentiation and has no specific immunohistological marker [1, 2]. Although UPS was defined in 2002, its epidemiology and prognosis has obscured because of its rarity. In reports of MFH, it commonly occurs in men and those older than 40 years, with a frequency of 1–2 cases per 100,000 people [3].MFH has been reported in limbs (68%), abdominal cavity/retroperitoneum (16%), trunk (9%), head and neck (3%), or gastrointestinal tract (4%). Its prognosis is poor, with a repoted 2-year survival rate of 60%, and a 5-year survival rate of 47% [3].

Primary gastric UPS or MFH is quite rare. Saito et al. reported 16 case reports of primary UPS or MFH of stomach, including one they reported and 14 found in PubMed using the keywords “undifferentiated pleomorphic sarcoma” and “malignant fibrous histiocytoma” during 1984–2011 [4–16]. The data of 16 cases and this case are summarized in Table 2. The average age was about 61 years old (range: 17–82 years old). Average diameter was 6.7 cm (range: 1–15 cm). Seven of the 16 patients showed invasion or metastasis and had died by the time they were reported. Only 4 of the 16 patients were 2-year survivors at the time they were reported. No 5-year survivors were yet reported. UPS is considered to have worse prognosis than other forms of MFH. In addition, there has been no report about gastric UPS producing G-CSF.

**Table 2:** Summarization in 16 cases diagnosed primary gastric UPS and MFH

No.	Year	Author	Age	Gender	Chief complaint	Site	Macroscopic forms	Size (cm)	Metastasis or invasion	Therapy	Outcome
1	1984	Morita	60	F	Pain	Antrum	SMT	3.5	Gall bladder	Operation, hemotherapy	Death, 6POM
2	1985	Shibuya	60	M	Bleeding	Antrum	Raised type	4.5	Ileum	Operation	Death, 3POM
3	1985	Randner	77	F	Pain	Body	SMT	1	Liver	None	Death
4	1988	Wright	42	M	Bleeding	Cardia	Ulcerative type	5	Lung	Operation, chemotherapy	Death, 17POM
5	1989	Ranthakrishman	51	M	Pain	Antrum	Tube-occupied type	Huge	None	Operation (stoma)	Death, 0.5POM
6	1998	Takagi	64	T	Amenia	Body	SMT	7.5	None	Operation	Alive, 6POM
7	1998	Wada	78	M	Pain	Body	Ulcerative type	5	None	Operation	Alive, 24POM
8	1998	Wada	77	M	Pain	Body	Ulcerative type	4	Lung	Operation	Death, 48POM
9	1998	Wierseme	37	F	Amenia	Body	Ulcerative type	5	None	Operation	Alive, 38POM
10	2000	Nakai	57	M	Pain	Antrum	Tube-occupied type	5	Liver	Operation, chemotherapy	Death, 7POM
11	2003	Accattatis	17	F	Pain	Body	Unknown	15	None	Operation	Death, 0.5POM
12	2006	Shinnshi	54	M	Pain	Antrum	SMT	4	None	Operation	Alive, 36POM
13	2007	Agaimy	79	M	None	Cardia	Ulcerative type	8	None	Operation	Death, 0.5POM
14	2007	Agaimy	68	F	Weight loss	Body	Extraluminal growth type	12	None	Operation	Alive, 6POM
15	2013	Kinoshita	74	F	Pain	body	Raised type	13	Liver	Chemotherapy	Death
16	2016	This case	82	M	Fever	Body	Raised type	8	None	Operation	Alive

*POM* postoperative months

According to the National Comprehensive Cancer Network (NCCN) Soft Tissue Sarcoma Guidelines, surgical resection with sufficient margin is the first-choice treatment for UPS. Amputation is required for tumors in limbs, so various surgical methods have been used with the aim of preserving function [17]. The first choice for resectable sarcomas in the inner trunk is considered to be en bloc excisions with ≥1-cm margins. Additional resection is recommended for margins narrower than 1 cm. As lymphatic metastasis is rare, systematic lymph node resection is not required. If hepatic metastases or infiltrations to nearby organs are resectable, their complete resection is desirable. Chemotherapy, radiation therapy, or hyperthermia have been used for unresectable tumors. The combination of cisplatin, ifosfamide, and adriamycin for reducing metastasis has been reported [18]. However, chemotherapy, radiation therapy, and hyperthermia have not led to satisfactory results [19, 20].

At first we suspected that infection or autoimmune diseases could have caused the fever and high CRP level; we had doubts about their association with the gastric tumor. However, as the fever and CRP level dramatically improved after the surgery, the gastric tumors were clearly related to the main complaints. There are reports about cases of UPS and MFH presenting initially with fever [21–23], and reports about cases of MFH producing actively cytokines such as G-CSF [24–27]. But. this is the first report about gastric UPS with fever and rapid growth due to producing G-CSF. G-CSF is thought to cause paraneoplastic syndrome such as fever or increased CRP, and cytokines such as G-CSF have been suggested as markers for UPS. In this case, we should reflect that treatment was delayed because we could not understand that these symptoms could be a clue to the diagnosis.

## Conclusions

We found a rare case of gastric UPS. As UPS is a rapidly growing, frequently recurring tumor, its prognosis is poor. Patients require strict postoperative observation.

## Abbreviations

(PET)GIST: Gastrointestinal stromal tumor
CRP: C-reactive protein
CT: Computed tomography
G-CSF: Granulocyte colony-stimulating factor
GIE: Gastrointestinal endoscopy
MFH: Malignant fibrous histiocytoma
NCCN: National Comprehensive Cancer Network
PET: Positron emission tomography
UPS: Undifferentiated high-grade pleomorphic sarcoma
WHO: World Health Organization
